# Supplementary material for: Associations between PM2.5 Components and Mortality of Ischemic Stroke, Chronic Obstructive Pulmonary Disease and Diabetes in Beijing, China
Source: Toxics. 2024 May 23;12(6):381. doi: 10.3390/toxics12060381 (PMC11209520; doi:10.3390/toxics12060381)
Supplement: Supplementary file 1 [file toxics-12-00381-s001.zip › toxics-2982569-SI.pdf]

Table S1. Relative risks (95%CI) for single-day lag and cumulative-day lag for IS, DM and COPD mortality

lag	SO_4^{2-}	NH_4^+	NO_3^-	OM	BC
IS					
lag 0	1.044 (0.996, 1.094)	1.031 (0.981, 1.083)	1.009 (0.976, 1.043)	1.003 (0.970, 1.038)	1.019 (0.877, 1.186)
lag 1	1.007 (0.963, 1.054)	1.012 (0.969, 1.057)	1.012 (0.984, 1.041)	1.020 (0.987, 1.053)	1.097 (0.944, 1.275)
lag 2	0.997 (0.971, 1.024)	1.001 (0.974, 1.028)	1.001 (0.984, 1.019)	0.993 (0.973, 1.012)	0.973 (0.887, 1.066)
lag 3	1.004 (0.986, 1.022)	1.005 (0.987, 1.023)	1.003 (0.991, 1.015)	1.000 (0.987, 1.014)	0.996 (0.935, 1.060)
lag 4	1.010 (0.994, 1.027)	1.010 (0.993, 1.027)	1.007 (0.995, 1.018)	1.011 (0.999, 1.024)	1.037 (0.979, 1.099)
lag 5	1.011 (0.994, 1.028)	1.011 (0.994, 1.027)	1.007 (0.996, 1.018)	1.011 (0.998, 1.023)	1.037 (0.980, 1.097)
lag 6	1.008 (0.995, 1.021)	1.008 (0.994, 1.021)	1.005 (0.996, 1.014)	1.002 (0.992, 1.012)	1.007 (0.962, 1.054)
lag 7	1.002 (0.977, 1.029)	1.003 (0.977, 1.029)	1.001 (0.984, 1.019)	0.990 (0.971, 1.009)	0.965 (0.883, 1.053)
lag 0-1	1.052 (1.000, 1.106)	1.044 (0.989, 1.101)	1.021 (0.984, 1.060)	1.023 (0.983, 1.065)	1.119 (0.942, 1.328)
lag 0-2	1.049 (0.999, 1.101)	1.044 (0.991, 1.101)	1.022 (0.986, 1.060)	1.016 (0.978, 1.055)	1.088 (0.922, 1.285)
lag 0-3	1.052 (0.998, 1.110)	1.050 (0.992, 1.111)	1.026 (0.986, 1.066)	1.016 (0.976, 1.057)	1.083 (0.908, 1.293)
lag 0-4	1.063 (1.005, 1.124)	1.060 (0.998, 1.126)	1.032 (0.991, 1.076)	1.027 (0.986, 1.071)	1.124 (0.933, 1.353)
lag 0-5	1.075 (1.011, 1.143)	1.072 (1.003, 1.144)	1.040 (0.994, 1.087)	1.038 (0.992, 1.086)	1.165 (0.951, 1.428)
lag 0-6	1.083 (1.014, 1.157)	1.080 (1.006, 1.159)	1.044 (0.996, 1.095)	1.040 (0.992, 1.091)	1.174 (0.945, 1.459)
lag 0-7	1.085 (1.010, 1.167)	1.083 (1.003, 1.169)	1.046 (0.993, 1.101)	1.030 (0.978, 1.084)	1.133 (0.894, 1.435)
DM					
lag 0	1.036 (0.960, 1.118)	1.018 (0.940, 1.102)	1.007 (0.954, 1.063)	1.036 (0.980, 1.094)	1.142 (0.895, 1.457)
lag 1	0.983 (0.914, 1.056)	1.001 (0.933, 1.073)	1.003 (0.959, 1.050)	1.015 (0.963, 1.070)	1.099 (0.863, 1.400)
lag 2	1.052 (1.007, 1.098)	1.051 (1.007, 1.097)	1.037 (1.008, 1.067)	1.038 (1.005, 1.071)	1.159 (0.998, 1.345)
lag 3	1.012 (0.983, 1.042)	1.007 (0.979, 1.037)	1.005 (0.986, 1.025)	1.009 (0.987, 1.032)	1.020 (0.922, 1.129)
lag 4	0.970 (0.944, 0.996)	0.967 (0.941, 0.993)	0.976 (0.958, 0.994)	0.983 (0.964, 1.004)	0.920 (0.837, 1.012)
lag 5	0.968 (0.943, 0.994)	0.965 (0.939, 0.991)	0.974 (0.957, 0.992)	0.981 (0.962, 1.001)	0.920 (0.839, 1.009)
lag 6	0.995 (0.974, 1.016)	0.989 (0.968, 1.011)	0.992 (0.977, 1.006)	0.995 (0.979, 1.011)	0.987 (0.916, 1.063)
lag 7	1.036 (0.994, 1.080)	1.028 (0.986, 1.072)	1.019 (0.992, 1.048)	1.017 (0.987, 1.048)	1.096 (0.951, 1.263)
lag 0-1	1.018 (0.939, 1.104)	1.018 (0.934, 1.110)	1.010 (0.952, 1.072)	1.052 (0.986, 1.121)	1.255 (0.953, 1.654)
lag 0-2	1.071 (0.990, 1.158)	1.070 (0.983, 1.165)	1.048 (0.988, 1.111)	1.091 (1.027, 1.160)	1.455 (1.116, 1.896)
lag 0-3	1.083 (0.995, 1.180)	1.078 (0.984, 1.181)	1.053 (0.989, 1.121)	1.101 (1.033, 1.173)	1.484 (1.118, 1.970)
lag 0-4	1.050 (0.960, 1.150)	1.042 (0.946, 1.148)	1.027 (0.962, 1.097)	1.083 (1.013, 1.157)	1.366 (1.013, 1.840)
lag 0-5	1.017 (0.921, 1.123)	1.005 (0.904, 1.118)	1.001 (0.931, 1.076)	1.063 (0.988, 1.143)	1.257 (0.905, 1.744)
lag 0-6	1.011 (0.909, 1.125)	0.995 (0.888, 1.114)	0.993 (0.919, 1.072)	1.057 (0.979, 1.142)	1.240 (0.872, 1.763)
lag 0-7	1.048 (0.932, 1.178)	1.023 (0.904, 1.157)	1.012 (0.931, 1.100)	1.075 (0.989, 1.169)	1.359 (0.927, 1.993)
COPD					
lag 0	1.063 (1.001, 1.128)	1.057 (0.994, 1.125)	1.026 (0.984, 1.070)	1.032 (0.989, 1.076)	1.228 (1.017, 1.482)
lag 1	0.993 (0.938, 1.051)	0.996 (0.943, 1.051)	1.002 (0.967, 1.038)	1.014 (0.975, 1.055)	1.000 (0.831, 1.204)
lag 2	0.986 (0.953, 1.020)	0.982 (0.949, 1.015)	0.988 (0.966, 1.010)	0.991 (0.967, 1.015)	0.962 (0.859, 1.077)
lag 3	1.004 (0.981, 1.027)	1.001 (0.978, 1.023)	0.999 (0.984, 1.014)	0.997 (0.981, 1.014)	1.001 (0.927, 1.082)
lag 4	1.018 (0.997, 1.039)	1.017 (0.996, 1.039)	1.010 (0.996, 1.024)	1.009 (0.994, 1.024)	1.043 (0.972, 1.120)

lag	SO_4^{2-}	NH_4^+	NO_3^-	OM	BC
lag 5	1.018 (0.998, 1.039)	1.019 (0.998, 1.040)	1.012 (0.998, 1.026)	1.013 (0.999, 1.028)	1.060 (0.990, 1.136)
lag 6	1.009 (0.993, 1.026)	1.010 (0.993, 1.027)	1.007 (0.996, 1.019)	1.013 (1.001, 1.026)	1.059 (1.001, 1.121)
lag 7	0.996 (0.964, 1.028)	0.996 (0.964, 1.029)	1.000 (0.978, 1.021)	1.011 (0.988, 1.034)	1.049 (0.942, 1.169)
lag 0-1	1.055 (0.990, 1.125)	1.053 (0.984, 1.127)	1.028 (0.981, 1.077)	1.047 (0.996, 1.099)	1.228 (0.991, 1.522)
lag 0-2	1.040 (0.978, 1.107)	1.033 (0.967, 1.104)	1.016 (0.970, 1.063)	1.037 (0.989, 1.087)	1.181 (0.960, 1.454)
lag 0-3	1.044 (0.976, 1.117)	1.034 (0.962, 1.111)	1.014 (0.966, 1.065)	1.034 (0.984, 1.086)	1.183 (0.949, 1.475)
lag 0-4	1.063 (0.990, 1.142)	1.052 (0.975, 1.135)	1.025 (0.973, 1.079)	1.043 (0.991, 1.098)	1.234 (0.978, 1.557)
lag 0-5	1.083 (1.002, 1.170)	1.071 (0.986, 1.164)	1.037 (0.980, 1.097)	1.057 (0.999, 1.117)	1.309 (1.015, 1.687)
lag 0-6	1.093 (1.005, 1.188)	1.082 (0.990, 1.183)	1.044 (0.984, 1.109)	1.071 (1.009, 1.136)	1.387 (1.057, 1.819)
lag 0-7	1.088 (0.993, 1.192)	1.078 (0.979, 1.187)	1.044 (0.979, 1.114)	1.082 (1.015, 1.154)	1.455 (1.082, 1.957)

Abbreviations: BC: black carbon; COPD: chronic obstructive pulmonary disease; CI: confidence interval; DM: diabetes mellitus; NH_4^+: ammonium ion; NO_3^-: nitrate ion; OM: organic matter; IS: Ischemic stroke; SD: standard deviation; SO_2: sulphur dioxide; SO_4^{2-}: sulfate ion.

Table S2. Relative risks (95%CI) for single-day lag and cumulative-day lag for IS, DM and COPD mortality in males and females

lag	SO_4^{2-}	NH_4^+	NO_3^-	SO_4^{2-}	NH_4^+
IS in male					
lag 0	1.044 (0.980, 1.112)	1.027 (0.961, 1.097)	1.007 (0.962, 1.053)	1.001 (0.956, 1.048)	1.018 (0.831, 1.246)
lag 1	1.007 (0.948, 1.069)	1.003 (0.946, 1.063)	1.009 (0.972, 1.049)	1.021 (0.977, 1.066)	1.096 (0.897, 1.341)
lag 2	0.993 (0.958, 1.030)	1.002 (0.967, 1.038)	1.002 (0.978, 1.026)	0.996 (0.970, 1.022)	0.990 (0.876, 1.119)
lag 3	1.009 (0.985, 1.034)	1.013 (0.989, 1.038)	1.007 (0.991, 1.023)	1.006 (0.988, 1.025)	1.035 (0.952, 1.125)
lag 4	1.021 (0.999, 1.044)	1.019 (0.996, 1.042)	1.012 (0.997, 1.027)	1.018 (1.001, 1.034)	1.082 (1.001, 1.168)
lag 5	1.017 (0.995, 1.040)	1.015 (0.993, 1.038)	1.010 (0.995, 1.025)	1.014 (0.998, 1.031)	1.061 (0.984, 1.143)
lag 6	1.002 (0.985, 1.020)	1.005 (0.987, 1.023)	1.004 (0.992, 1.016)	1.001 (0.988, 1.014)	0.996 (0.936, 1.059)
lag 7	0.983 (0.949, 1.017)	0.991 (0.957, 1.026)	0.996 (0.973, 1.020)	0.983 (0.958, 1.008)	0.915 (0.813, 1.030)
lag 0-1	1.051 (0.983, 1.125)	1.029 (0.957, 1.107)	1.016 (0.967, 1.068)	1.022 (0.969, 1.078)	1.116 (0.886, 1.406)
lag 0-2	1.044 (0.978, 1.115)	1.031 (0.961, 1.107)	1.017 (0.969, 1.069)	1.017 (0.967, 1.071)	1.105 (0.884, 1.381)
lag 0-3	1.054 (0.981, 1.131)	1.044 (0.968, 1.127)	1.024 (0.972, 1.080)	1.024 (0.970, 1.080)	1.144 (0.902, 1.451)
lag 0-4	1.076 (0.998, 1.160)	1.064 (0.982, 1.154)	1.036 (0.981, 1.095)	1.042 (0.985, 1.101)	1.237 (0.964, 1.588)
lag 0-5	1.094 (1.008, 1.188)	1.080 (0.989, 1.180)	1.047 (0.986, 1.112)	1.056 (0.994, 1.122)	1.312 (0.998, 1.725)
lag 0-6	1.097 (1.004, 1.198)	1.085 (0.987, 1.193)	1.051 (0.986, 1.121)	1.057 (0.991, 1.128)	1.306 (0.975, 1.750)
lag 0-7	1.078 (0.978, 1.188)	1.075 (0.970, 1.192)	1.047 (0.977, 1.123)	1.039 (0.969, 1.114)	1.195 (0.869, 1.643)
COPD in male					
lag 0	1.094 (1.009, 1.186)	1.086 (0.998, 1.181)	1.048 (0.990, 1.109)	1.056 (0.997, 1.118)	1.295 (1.002, 1.674)
lag 1	0.939 (0.869, 1.015)	0.948 (0.880, 1.020)	0.972 (0.926, 1.020)	0.974 (0.922, 1.029)	0.841 (0.652, 1.084)
lag 2	0.968 (0.924, 1.013)	0.964 (0.921, 1.009)	0.978 (0.949, 1.008)	0.982 (0.950, 1.014)	0.926 (0.793, 1.081)
lag 3	1.006 (0.976, 1.038)	1.002 (0.972, 1.034)	1.000 (0.980, 1.021)	1.002 (0.979, 1.025)	1.023 (0.920, 1.137)
lag 4	1.025 (0.996, 1.054)	1.023 (0.995, 1.053)	1.013 (0.994, 1.032)	1.014 (0.993, 1.035)	1.071 (0.972, 1.180)
lag 5	1.020 (0.992, 1.049)	1.019 (0.991, 1.048)	1.010 (0.992, 1.029)	1.014 (0.994, 1.035)	1.068 (0.972, 1.174)
lag 6	0.999 (0.977, 1.022)	0.997 (0.975, 1.021)	0.998 (0.983, 1.013)	1.006 (0.990, 1.023)	1.032 (0.954, 1.115)
lag 7	0.972 (0.929, 1.016)	0.968 (0.926, 1.012)	0.980 (0.952, 1.010)	0.995 (0.964, 1.027)	0.980 (0.845, 1.138)
lag 0-1	1.028 (0.943, 1.120)	1.029 (0.938, 1.128)	1.018 (0.956, 1.085)	1.028 (0.961, 1.099)	1.089 (0.813, 1.460)
lag 0-2	0.994 (0.914, 1.082)	0.992 (0.906, 1.086)	0.996 (0.935, 1.060)	1.009 (0.946, 1.076)	1.009 (0.759, 1.339)
lag 0-3	1.001 (0.913, 1.097)	0.994 (0.902, 1.096)	0.996 (0.931, 1.064)	1.010 (0.945, 1.081)	1.032 (0.762, 1.396)
lag 0-4	1.026 (0.931, 1.130)	1.017 (0.917, 1.128)	1.008 (0.940, 1.082)	1.024 (0.955, 1.099)	1.105 (0.804, 1.518)
lag 0-5	1.046 (0.941, 1.163)	1.036 (0.926, 1.160)	1.019 (0.944, 1.100)	1.039 (0.962, 1.121)	1.180 (0.834, 1.671)
lag 0-6	1.045 (0.933, 1.171)	1.034 (0.916, 1.167)	1.017 (0.937, 1.103)	1.045 (0.964, 1.134)	1.217 (0.839, 1.766)
lag 0-7	1.016 (0.897, 1.151)	1.001 (0.877, 1.142)	0.997 (0.912, 1.089)	1.040 (0.952, 1.136)	1.193 (0.795, 1.791)
DM in male					
lag 0	1.007 (0.901, 1.127)	0.971 (0.864, 1.092)	0.967 (0.893, 1.047)	1.011 (0.933, 1.095)	1.020 (0.714, 1.456)
lag 1	1.052 (0.947, 1.169)	1.073 (0.969, 1.188)	1.052 (0.984, 1.124)	1.088 (1.008, 1.175)	1.551 (1.090, 2.208)
lag 2	1.034 (0.970, 1.102)	1.035 (0.973, 1.102)	1.027 (0.985, 1.071)	1.029 (0.981, 1.078)	1.051 (0.844, 1.309)
lag 3	0.992 (0.951, 1.035)	0.987 (0.946, 1.029)	0.990 (0.963, 1.019)	0.993 (0.961, 1.026)	0.915 (0.788, 1.063)

lag	SO_4^{2-}	NH_4^+	NO_3^-	SO_4^{2-}	NH_4^+
lag 4	0.967 (0.930, 1.006)	0.962 (0.924, 1.001)	0.971 (0.946, 0.998)	0.980 (0.951, 1.010)	0.910 (0.792, 1.046)
lag 5	0.968 (0.931, 1.007)	0.964 (0.927, 1.003)	0.973 (0.948, 0.999)	0.982 (0.953, 1.010)	0.935 (0.816, 1.072)
lag 6	0.986 (0.956, 1.017)	0.985 (0.954, 1.016)	0.989 (0.968, 1.010)	0.992 (0.969, 1.016)	0.982 (0.880, 1.095)
lag 7	1.014 (0.955, 1.077)	1.015 (0.955, 1.078)	1.012 (0.972, 1.054)	1.008 (0.965, 1.054)	1.041 (0.846, 1.282)
lag 0-1	1.060 (0.942, 1.193)	1.042 (0.919, 1.182)	1.017 (0.933, 1.110)	1.100 (1.002, 1.207)	1.581 (1.060, 2.360)
lag 0-2	1.096 (0.978, 1.229)	1.079 (0.954, 1.221)	1.045 (0.959, 1.138)	1.132 (1.036, 1.236)	1.662 (1.132, 2.439)
lag 0-3	1.088 (0.960, 1.232)	1.065 (0.932, 1.216)	1.035 (0.945, 1.134)	1.123 (1.024, 1.232)	1.521 (1.008, 2.295)
lag 0-4	1.052 (0.922, 1.200)	1.024 (0.890, 1.180)	1.005 (0.913, 1.107)	1.101 (1.000, 1.213)	1.384 (0.897, 2.135)
lag 0-5	1.018 (0.881, 1.177)	0.988 (0.846, 1.153)	0.978 (0.881, 1.086)	1.081 (0.973, 1.201)	1.294 (0.803, 2.085)
lag 0-6	1.004 (0.859, 1.174)	0.972 (0.824, 1.148)	0.967 (0.864, 1.082)	1.073 (0.958, 1.200)	1.270 (0.761, 2.120)
lag 0-7	1.019 (0.859, 1.209)	0.987 (0.824, 1.181)	0.979 (0.867, 1.106)	1.081 (0.958, 1.221)	1.323 (0.758, 2.309)
IS in female					
lag 0	1.045 (0.975, 1.120)	1.037 (0.965, 1.115)	1.011 (0.963, 1.062)	1.007 (0.958, 1.058)	1.027 (0.823, 1.280)
lag 1	1.009 (0.944, 1.077)	1.025 (0.962, 1.092)	1.016 (0.975, 1.059)	1.019 (0.972, 1.069)	1.103 (0.885, 1.374)
lag 2	1.002 (0.964, 1.043)	1.000 (0.961, 1.040)	1.002 (0.976, 1.028)	0.990 (0.961, 1.019)	0.954 (0.833, 1.092)
lag 3	0.997 (0.971, 1.024)	0.995 (0.969, 1.022)	0.999 (0.981, 1.016)	0.993 (0.974, 1.013)	0.949 (0.866, 1.041)
lag 4	0.996 (0.973, 1.021)	0.999 (0.975, 1.024)	1.000 (0.984, 1.017)	1.003 (0.985, 1.022)	0.984 (0.904, 1.072)
lag 5	1.003 (0.979, 1.027)	1.005 (0.981, 1.029)	1.003 (0.987, 1.019)	1.006 (0.988, 1.024)	1.008 (0.927, 1.095)
lag 6	1.014 (0.995, 1.033)	1.011 (0.992, 1.031)	1.005 (0.992, 1.019)	1.004 (0.989, 1.018)	1.021 (0.955, 1.092)
lag 7	1.028 (0.990, 1.067)	1.019 (0.981, 1.058)	1.008 (0.983, 1.034)	0.999 (0.972, 1.027)	1.030 (0.906, 1.172)
lag 0-1	1.054 (0.979, 1.134)	1.063 (0.983, 1.149)	1.028 (0.973, 1.085)	1.026 (0.968, 1.087)	1.132 (0.881, 1.455)
lag 0-2	1.056 (0.983, 1.134)	1.062 (0.983, 1.148)	1.029 (0.976, 1.086)	1.015 (0.961, 1.073)	1.080 (0.847, 1.375)
lag 0-3	1.053 (0.974, 1.138)	1.057 (0.973, 1.149)	1.028 (0.971, 1.088)	1.009 (0.952, 1.068)	1.025 (0.792, 1.327)
lag 0-4	1.049 (0.967, 1.139)	1.056 (0.967, 1.153)	1.028 (0.968, 1.092)	1.012 (0.953, 1.074)	1.009 (0.770, 1.323)
lag 0-5	1.052 (0.962, 1.151)	1.061 (0.964, 1.168)	1.031 (0.966, 1.100)	1.018 (0.953, 1.087)	1.017 (0.756, 1.367)
lag 0-6	1.067 (0.969, 1.175)	1.073 (0.968, 1.190)	1.036 (0.966, 1.111)	1.022 (0.953, 1.095)	1.038 (0.756, 1.426)
lag 0-7	1.096 (0.986, 1.219)	1.093 (0.978, 1.223)	1.044 (0.968, 1.127)	1.021 (0.947, 1.101)	1.070 (0.757, 1.511)
COPD in female					
lag 0	1.030 (0.942, 1.126)	1.026 (0.935, 1.126)	1.001 (0.940, 1.065)	1.007 (0.946, 1.072)	1.168 (0.883, 1.544)
lag 1	1.059 (0.974, 1.152)	1.055 (0.973, 1.143)	1.038 (0.985, 1.094)	1.061 (1.001, 1.125)	1.225 (0.931, 1.613)
lag 2	1.010 (0.960, 1.061)	1.005 (0.957, 1.056)	1.001 (0.969, 1.034)	1.001 (0.967, 1.037)	1.004 (0.850, 1.186)
lag 3	1.002 (0.969, 1.037)	1.000 (0.967, 1.034)	0.998 (0.976, 1.021)	0.993 (0.969, 1.017)	0.976 (0.870, 1.096)
lag 4	1.010 (0.979, 1.041)	1.010 (0.979, 1.041)	1.007 (0.986, 1.028)	1.003 (0.981, 1.025)	1.012 (0.911, 1.124)
lag 5	1.016 (0.985, 1.047)	1.018 (0.987, 1.050)	1.014 (0.993, 1.034)	1.012 (0.991, 1.034)	1.051 (0.949, 1.163)
lag 6	1.021 (0.997, 1.046)	1.026 (1.000, 1.051)	1.019 (1.002, 1.036)	1.021 (1.003, 1.039)	1.091 (1.004, 1.186)
lag 7	1.026 (0.978, 1.076)	1.032 (0.984, 1.083)	1.024 (0.992, 1.057)	1.029 (0.995, 1.064)	1.133 (0.967, 1.328)
lag 0-1	1.091 (0.993, 1.200)	1.082 (0.978, 1.197)	1.039 (0.970, 1.113)	1.069 (0.994, 1.150)	1.431 (1.041, 1.967)
lag 0-2	1.102 (1.005, 1.208)	1.088 (0.985, 1.201)	1.040 (0.972, 1.113)	1.070 (0.998, 1.147)	1.437 (1.057, 1.953)
lag 0-3	1.104 (0.999, 1.220)	1.087 (0.978, 1.210)	1.038 (0.966, 1.117)	1.063 (0.988, 1.142)	1.402 (1.011, 1.945)

lag	SO_4^{2-}	NH_4^+	NO_3^-	SO_4^{2-}	NH_4^+
lag 0-4	1.115 (1.003, 1.239)	1.098 (0.981, 1.229)	1.045 (0.968, 1.129)	1.066 (0.988, 1.149)	1.419 (1.006, 2.002)
lag 0-5	1.132 (1.009, 1.271)	1.118 (0.988, 1.264)	1.060 (0.975, 1.152)	1.079 (0.993, 1.171)	1.491 (1.024, 2.172)
lag 0-6	1.156 (1.021, 1.309)	1.146 (1.005, 1.308)	1.080 (0.988, 1.180)	1.101 (1.009, 1.201)	1.626 (1.088, 2.432)
lag 0-7	1.186 (1.036, 1.359)	1.183 (1.026, 1.365)	1.106 (1.005, 1.217)	1.133 (1.031, 1.245)	1.843 (1.190, 2.854)
DM in female					
lag 0	1.060 (0.954, 1.177)	1.057 (0.947, 1.180)	1.041 (0.966, 1.121)	1.056 (0.979, 1.139)	1.248 (0.893, 1.745)
lag 1	0.928 (0.840, 1.026)	0.944 (0.858, 1.039)	0.965 (0.906, 1.028)	0.958 (0.891, 1.030)	0.828 (0.593, 1.155)
lag 2	1.066 (1.005, 1.131)	1.063 (1.002, 1.128)	1.045 (1.005, 1.087)	1.045 (1.000, 1.092)	1.254 (1.023, 1.538)
lag 3	1.028 (0.988, 1.070)	1.025 (0.985, 1.066)	1.018 (0.991, 1.045)	1.023 (0.992, 1.054)	1.116 (0.971, 1.282)
lag 4	0.972 (0.937, 1.008)	0.971 (0.936, 1.008)	0.980 (0.955, 1.005)	0.987 (0.960, 1.014)	0.931 (0.818, 1.060)
lag 5	0.969 (0.934, 1.005)	0.966 (0.931, 1.003)	0.976 (0.952, 1.000)	0.982 (0.956, 1.009)	0.910 (0.802, 1.033)
lag 6	1.002 (0.973, 1.031)	0.994 (0.965, 1.024)	0.995 (0.975, 1.015)	0.998 (0.976, 1.020)	0.993 (0.897, 1.099)
lag 7	1.055 (0.997, 1.116)	1.040 (0.982, 1.102)	1.026 (0.987, 1.066)	1.025 (0.983, 1.068)	1.144 (0.941, 1.390)
lag 0-1	0.984 (0.880, 1.100)	0.998 (0.886, 1.125)	1.004 (0.925, 1.090)	1.012 (0.926, 1.106)	1.033 (0.705, 1.514)
lag 0-2	1.049 (0.940, 1.169)	1.061 (0.944, 1.193)	1.049 (0.968, 1.138)	1.057 (0.972, 1.151)	1.296 (0.896, 1.874)
lag 0-3	1.078 (0.958, 1.213)	1.087 (0.958, 1.234)	1.068 (0.979, 1.164)	1.081 (0.990, 1.181)	1.446 (0.976, 2.143)
lag 0-4	1.048 (0.925, 1.187)	1.056 (0.924, 1.207)	1.046 (0.955, 1.146)	1.067 (0.973, 1.170)	1.346 (0.890, 2.036)
lag 0-5	1.016 (0.886, 1.164)	1.020 (0.881, 1.181)	1.021 (0.924, 1.128)	1.048 (0.947, 1.159)	1.225 (0.778, 1.930)
lag 0-6	1.017 (0.878, 1.179)	1.014 (0.867, 1.187)	1.015 (0.913, 1.130)	1.045 (0.939, 1.164)	1.217 (0.747, 1.980)
lag 0-7	1.073 (0.913, 1.261)	1.055 (0.889, 1.252)	1.042 (0.928, 1.170)	1.071 (0.954, 1.203)	1.391 (0.819, 2.363)

Abbreviations: BC: black carbon; COPD: chronic obstructive pulmonary disease; CI: confidence interval; DM: diabetes mellitus; NH_4^+: ammonium ion; NO_3^-: nitrate ion; OM: organic matter; IS: Ischemic stroke; SD: standard deviation; SO_2: sulphur dioxide; SO_4^{2-}: sulfate ion.

Table S3. Relative risks (95%CI) for single-day lag and cumulative-day lag for IS, DM and COPD mortality in elderly population (age ≥ 65 years old) and the younger population (age < 65 years old)

lag	SO_4^{2-}	NH_4^+	NO_3^-	OM	BC
IS in elder					
lag 0	1.034 (0.983, 1.087)	1.020 (0.968, 1.075)	1.002 (0.967, 1.039)	0.997 (0.962, 1.034)	0.982 (0.835, 1.154)
lag 1	1.016 (0.968, 1.066)	1.018 (0.972, 1.066)	1.014 (0.983, 1.045)	1.025 (0.991, 1.062)	1.126 (0.960, 1.322)
lag 2	0.992 (0.964, 1.021)	0.997 (0.969, 1.026)	0.999 (0.980, 1.018)	0.990 (0.969, 1.011)	0.960 (0.870, 1.059)
lag 3	1.001 (0.982, 1.020)	1.003 (0.984, 1.023)	1.003 (0.990, 1.015)	0.998 (0.984, 1.013)	0.983 (0.919, 1.051)
lag 4	1.012 (0.994, 1.030)	1.012 (0.994, 1.030)	1.008 (0.996, 1.020)	1.012 (0.999, 1.025)	1.036 (0.973, 1.102)
lag 5	1.013 (0.996, 1.031)	1.012 (0.995, 1.030)	1.008 (0.996, 1.020)	1.012 (0.999, 1.025)	1.039 (0.978, 1.104)
lag 6	1.008 (0.994, 1.022)	1.007 (0.993, 1.022)	1.004 (0.995, 1.014)	1.003 (0.993, 1.014)	1.008 (0.960, 1.059)
lag 7	0.999 (0.972, 1.027)	0.999 (0.972, 1.027)	0.999 (0.980, 1.017)	0.990 (0.970, 1.010)	0.962 (0.876, 1.057)
lag 0-1	1.051 (0.996, 1.108)	1.038 (0.980, 1.100)	1.016 (0.976, 1.057)	1.023 (0.980, 1.067)	1.106 (0.920, 1.329)
lag 0-2	1.043 (0.989, 1.098)	1.036 (0.979, 1.096)	1.015 (0.976, 1.055)	1.012 (0.972, 1.054)	1.061 (0.888, 1.267)
lag 0-3	1.044 (0.986, 1.105)	1.039 (0.978, 1.104)	1.018 (0.976, 1.061)	1.011 (0.969, 1.054)	1.043 (0.863, 1.260)
lag 0-4	1.056 (0.995, 1.122)	1.051 (0.986, 1.121)	1.026 (0.982, 1.072)	1.023 (0.978, 1.069)	1.080 (0.885, 1.318)
lag 0-5	1.071 (1.003, 1.143)	1.064 (0.992, 1.142)	1.034 (0.986, 1.085)	1.035 (0.986, 1.086)	1.122 (0.903, 1.395)
lag 0-6	1.079 (1.006, 1.158)	1.072 (0.994, 1.156)	1.039 (0.987, 1.093)	1.038 (0.986, 1.093)	1.132 (0.897, 1.428)
lag 0-7	1.078 (0.998, 1.165)	1.071 (0.987, 1.162)	1.037 (0.981, 1.096)	1.028 (0.972, 1.086)	1.088 (0.845, 1.403)
COPD in elder					
lag 0	1.069 (1.005, 1.137)	1.065 (0.999, 1.136)	1.030 (0.986, 1.075)	1.038 (0.993, 1.084)	1.257 (1.035, 1.527)
lag 1	0.981 (0.925, 1.040)	0.983 (0.930, 1.040)	0.995 (0.959, 1.032)	1.010 (0.969, 1.052)	0.974 (0.804, 1.181)
lag 2	0.996 (0.962, 1.031)	0.993 (0.959, 1.027)	0.995 (0.972, 1.017)	0.995 (0.971, 1.020)	0.993 (0.883, 1.115)
lag 3	1.009 (0.985, 1.032)	1.006 (0.983, 1.030)	1.002 (0.987, 1.018)	0.999 (0.982, 1.017)	1.017 (0.939, 1.102)
lag 4	1.014 (0.992, 1.036)	1.014 (0.992, 1.036)	1.008 (0.993, 1.022)	1.007 (0.991, 1.023)	1.036 (0.963, 1.114)
lag 5	1.014 (0.992, 1.035)	1.014 (0.993, 1.036)	1.009 (0.995, 1.023)	1.011 (0.996, 1.027)	1.053 (0.980, 1.131)
lag 6	1.010 (0.993, 1.027)	1.011 (0.993, 1.028)	1.007 (0.996, 1.019)	1.014 (1.001, 1.026)	1.069 (1.008, 1.133)
lag 7	1.005 (0.972, 1.039)	1.005 (0.972, 1.039)	1.004 (0.982, 1.027)	1.015 (0.991, 1.039)	1.084 (0.970, 1.213)
lag 0-1	1.049 (0.982, 1.120)	1.047 (0.976, 1.123)	1.025 (0.976, 1.075)	1.048 (0.996, 1.103)	1.225 (0.982, 1.529)
lag 0-2	1.044 (0.979, 1.113)	1.039 (0.970, 1.113)	1.019 (0.972, 1.069)	1.043 (0.993, 1.095)	1.216 (0.982, 1.507)
lag 0-3	1.053 (0.982, 1.129)	1.046 (0.971, 1.126)	1.021 (0.971, 1.074)	1.042 (0.991, 1.097)	1.237 (0.985, 1.555)
lag 0-4	1.067 (0.992, 1.149)	1.060 (0.980, 1.147)	1.029 (0.976, 1.086)	1.050 (0.995, 1.107)	1.281 (1.008, 1.629)
lag 0-5	1.082 (0.998, 1.172)	1.075 (0.987, 1.171)	1.038 (0.980, 1.101)	1.061 (1.002, 1.124)	1.349 (1.037, 1.754)
lag 0-6	1.093 (1.002, 1.191)	1.087 (0.991, 1.191)	1.046 (0.983, 1.113)	1.076 (1.012, 1.144)	1.441 (1.088, 1.909)
lag 0-7	1.098 (0.999, 1.207)	1.092 (0.988, 1.207)	1.050 (0.982, 1.123)	1.092 (1.022, 1.167)	1.563 (1.150, 2.124)
DM in elder					
lag 0	0.995 (0.908, 1.089)	0.984 (0.895, 1.081)	0.988 (0.927, 1.053)	1.008 (0.945, 1.076)	1.025 (0.769, 1.368)
lag 1	0.982 (0.901, 1.069)	0.994 (0.915, 1.080)	1.000 (0.947, 1.055)	1.023 (0.962, 1.089)	1.141 (0.857, 1.518)
lag 2	1.058 (1.005, 1.113)	1.053 (1.001, 1.108)	1.037 (1.002, 1.072)	1.035 (0.997, 1.076)	1.142 (0.957, 1.364)
lag 3	1.012 (0.978, 1.047)	1.008 (0.974, 1.043)	1.005 (0.982, 1.028)	1.007 (0.981, 1.034)	1.008 (0.894, 1.137)

lag	SO_4^{2-}	NH_4^+	NO_3^-	OM	BC
lag 4	0.963 (0.933, 0.994)	0.963 (0.932, 0.994)	0.974 (0.953, 0.995)	0.983 (0.959, 1.007)	0.915 (0.818, 1.024)
lag 5	0.958 (0.928, 0.989)	0.957 (0.927, 0.987)	0.970 (0.949, 0.990)	0.978 (0.955, 1.001)	0.901 (0.807, 1.006)
lag 6	0.982 (0.958, 1.007)	0.977 (0.952, 1.002)	0.984 (0.967, 1.001)	0.985 (0.967, 1.004)	0.936 (0.857, 1.023)
lag 7	1.023 (0.974, 1.074)	1.011 (0.962, 1.062)	1.007 (0.975, 1.041)	1.000 (0.964, 1.036)	1.000 (0.844, 1.184)
lag 0-1	0.976 (0.887, 1.075)	0.978 (0.883, 1.083)	0.988 (0.920, 1.060)	1.032 (0.956, 1.113)	1.170 (0.844, 1.623)
lag 0-2	1.033 (0.941, 1.134)	1.030 (0.931, 1.139)	1.024 (0.955, 1.098)	1.068 (0.994, 1.149)	1.337 (0.976, 1.831)
lag 0-3	1.045 (0.944, 1.156)	1.038 (0.931, 1.156)	1.029 (0.955, 1.108)	1.076 (0.997, 1.161)	1.347 (0.963, 1.886)
lag 0-4	1.006 (0.904, 1.120)	0.999 (0.891, 1.120)	1.002 (0.926, 1.083)	1.057 (0.977, 1.145)	1.233 (0.866, 1.756)
lag 0-5	0.964 (0.857, 1.084)	0.955 (0.843, 1.083)	0.971 (0.891, 1.058)	1.034 (0.948, 1.127)	1.110 (0.752, 1.638)
lag 0-6	0.947 (0.834, 1.075)	0.933 (0.816, 1.068)	0.955 (0.872, 1.047)	1.019 (0.929, 1.117)	1.039 (0.685, 1.578)
lag 0-7	0.968 (0.843, 1.112)	0.943 (0.815, 1.092)	0.962 (0.871, 1.063)	1.018 (0.922, 1.125)	1.040 (0.660, 1.638)
IS in younger					
lag 0	1.113 (0.980, 1.264)	1.108 (0.969, 1.267)	1.054 (0.962, 1.154)	1.043 (0.951, 1.144)	1.306 (0.870, 1.962)
lag 1	0.952 (0.842, 1.077)	0.975 (0.866, 1.098)	1.002 (0.927, 1.084)	0.981 (0.898, 1.073)	0.919 (0.611, 1.384)
lag 2	1.027 (0.955, 1.104)	1.023 (0.951, 1.099)	1.016 (0.968, 1.066)	1.010 (0.957, 1.066)	1.058 (0.827, 1.355)
lag 3	1.020 (0.972, 1.071)	1.016 (0.967, 1.067)	1.007 (0.974, 1.041)	1.014 (0.977, 1.052)	1.079 (0.912, 1.277)
lag 4	0.997 (0.954, 1.042)	1.000 (0.956, 1.046)	0.998 (0.968, 1.029)	1.007 (0.975, 1.041)	1.049 (0.900, 1.222)
lag 5	0.995 (0.952, 1.039)	1.000 (0.957, 1.046)	0.999 (0.970, 1.029)	1.001 (0.969, 1.034)	1.024 (0.881, 1.190)
lag 6	1.006 (0.972, 1.042)	1.012 (0.976, 1.049)	1.007 (0.983, 1.032)	0.996 (0.970, 1.023)	1.005 (0.889, 1.136)
lag 7	1.025 (0.957, 1.099)	1.029 (0.959, 1.103)	1.019 (0.972, 1.068)	0.991 (0.942, 1.042)	0.987 (0.779, 1.251)
lag 0-1	1.060 (0.926, 1.213)	1.081 (0.934, 1.250)	1.056 (0.955, 1.168)	1.024 (0.920, 1.140)	1.201 (0.758, 1.901)
lag 0-2	1.088 (0.954, 1.241)	1.105 (0.958, 1.275)	1.073 (0.972, 1.185)	1.034 (0.933, 1.146)	1.271 (0.816, 1.978)
lag 0-3	1.110 (0.963, 1.281)	1.123 (0.963, 1.309)	1.081 (0.972, 1.201)	1.048 (0.942, 1.167)	1.372 (0.856, 2.199)
lag 0-4	1.107 (0.952, 1.287)	1.123 (0.954, 1.321)	1.078 (0.964, 1.206)	1.056 (0.944, 1.181)	1.438 (0.877, 2.360)
lag 0-5	1.101 (0.933, 1.299)	1.123 (0.940, 1.342)	1.077 (0.954, 1.216)	1.057 (0.936, 1.194)	1.473 (0.858, 2.531)
lag 0-6	1.108 (0.928, 1.323)	1.136 (0.939, 1.374)	1.085 (0.953, 1.235)	1.053 (0.925, 1.198)	1.480 (0.829, 2.642)
lag 0-7	1.136 (0.935, 1.380)	1.168 (0.950, 1.437)	1.106 (0.961, 1.273)	1.043 (0.906, 1.201)	1.461 (0.775, 2.754)
COPD in younger					
lag 0	1.003 (0.789, 1.274)	0.969 (0.755, 1.243)	0.984 (0.832, 1.163)	0.972 (0.823, 1.147)	0.956 (0.452, 2.023)
lag 1	1.170 (0.929, 1.474)	1.174 (0.943, 1.462)	1.096 (0.951, 1.264)	1.045 (0.893, 1.223)	1.331 (0.635, 2.791)
lag 2	0.846 (0.736, 0.972)	0.835 (0.729, 0.957)	0.897 (0.820, 0.981)	0.925 (0.842, 1.017)	0.600 (0.381, 0.943)
lag 3	0.942 (0.859, 1.034)	0.925 (0.843, 1.014)	0.953 (0.897, 1.013)	0.973 (0.911, 1.039)	0.804 (0.588, 1.099)
lag 4	1.090 (1.003, 1.183)	1.073 (0.987, 1.165)	1.043 (0.988, 1.102)	1.036 (0.978, 1.098)	1.172 (0.886, 1.549)
lag 5	1.094 (1.009, 1.187)	1.086 (1.001, 1.178)	1.055 (1.000, 1.113)	1.042 (0.985, 1.102)	1.182 (0.902, 1.550)
lag 6	0.997 (0.934, 1.065)	1.001 (0.936, 1.070)	1.010 (0.966, 1.056)	1.006 (0.960, 1.054)	0.927 (0.740, 1.161)
lag 7	0.866 (0.758, 0.988)	0.881 (0.773, 1.003)	0.941 (0.864, 1.025)	0.952 (0.871, 1.041)	0.640 (0.416, 0.986)
lag 0-1	1.173 (0.906, 1.519)	1.137 (0.863, 1.497)	1.079 (0.893, 1.302)	1.015 (0.835, 1.235)	1.273 (0.536, 3.022)
lag 0-2	0.992 (0.773, 1.274)	0.949 (0.725, 1.243)	0.967 (0.804, 1.164)	0.940 (0.780, 1.133)	0.763 (0.331, 1.758)
lag 0-3	0.935 (0.713, 1.226)	0.878 (0.657, 1.173)	0.922 (0.757, 1.124)	0.914 (0.753, 1.110)	0.614 (0.254, 1.486)

lag	SO_4^{2-}	NH_4^+	NO_3^-	OM	BC
lag 0-4	1.019 (0.766, 1.356)	0.942 (0.693, 1.279)	0.962 (0.781, 1.185)	0.947 (0.774, 1.160)	0.719 (0.285, 1.818)
lag 0-5	1.115 (0.817, 1.522)	1.022 (0.732, 1.427)	1.015 (0.810, 1.272)	0.987 (0.793, 1.228)	0.850 (0.311, 2.328)
lag 0-6	1.112 (0.797, 1.550)	1.023 (0.717, 1.461)	1.025 (0.806, 1.303)	0.993 (0.787, 1.251)	0.788 (0.270, 2.301)
lag 0-7	0.962 (0.668, 1.386)	0.901 (0.612, 1.327)	0.964 (0.744, 1.250)	0.945 (0.737, 1.213)	0.505 (0.157, 1.620)
DM in younger					
lag 0	1.184 (1.015, 1.382)	1.139 (0.969, 1.339)	1.073 (0.962, 1.197)	1.128 (1.011, 1.260)	1.618 (0.990, 2.646)
lag 1	0.984 (0.850, 1.140)	1.020 (0.885, 1.174)	1.012 (0.923, 1.110)	0.989 (0.889, 1.099)	0.976 (0.597, 1.595)
lag 2	1.032 (0.945, 1.127)	1.042 (0.956, 1.137)	1.038 (0.980, 1.099)	1.041 (0.976, 1.110)	1.196 (0.887, 1.614)
lag 3	1.013 (0.955, 1.075)	1.007 (0.949, 1.069)	1.007 (0.969, 1.048)	1.015 (0.971, 1.061)	1.061 (0.863, 1.305)
lag 4	0.993 (0.940, 1.048)	0.982 (0.929, 1.038)	0.983 (0.948, 1.020)	0.988 (0.949, 1.029)	0.949 (0.785, 1.147)
lag 5	1.003 (0.950, 1.058)	0.993 (0.941, 1.049)	0.990 (0.955, 1.026)	0.995 (0.957, 1.035)	0.995 (0.827, 1.198)
lag 6	1.034 (0.991, 1.079)	1.031 (0.986, 1.077)	1.017 (0.988, 1.048)	1.026 (0.994, 1.059)	1.164 (1.004, 1.351)
lag 7	1.078 (0.993, 1.172)	1.083 (0.996, 1.177)	1.057 (0.999, 1.117)	1.070 (1.008, 1.136)	1.438 (1.086, 1.904)
lag 0-1	1.165 (0.991, 1.371)	1.161 (0.975, 1.383)	1.086 (0.964, 1.225)	1.116 (0.982, 1.268)	1.579 (0.906, 2.750)
lag 0-2	1.203 (1.027, 1.409)	1.210 (1.020, 1.436)	1.127 (1.002, 1.268)	1.161 (1.029, 1.312)	1.889 (1.108, 3.220)
lag 0-3	1.218 (1.025, 1.448)	1.219 (1.014, 1.467)	1.135 (1.001, 1.288)	1.179 (1.039, 1.338)	2.004 (1.133, 3.544)
lag 0-4	1.209 (1.007, 1.452)	1.197 (0.984, 1.457)	1.117 (0.978, 1.275)	1.165 (1.020, 1.331)	1.902 (1.042, 3.472)
lag 0-5	1.213 (0.992, 1.483)	1.190 (0.960, 1.475)	1.105 (0.956, 1.278)	1.160 (1.003, 1.341)	1.893 (0.977, 3.669)
lag 0-6	1.254 (1.010, 1.558)	1.226 (0.974, 1.544)	1.125 (0.963, 1.313)	1.190 (1.019, 1.389)	2.204 (1.084, 4.482)
lag 0-7	1.353 (1.067, 1.715)	1.327 (1.033, 1.705)	1.188 (1.005, 1.406)	1.273 (1.077, 1.504)	3.169 (1.467, 6.847)

Abbreviations: BC: black carbon; COPD: chronic obstructive pulmonary disease; CI: confidence interval; DM: diabetes mellitus; NH_4^+: ammonium ion; NO_3^-: nitrate ion; OM: organic matter; IS: Ischemic stroke; SD: standard deviation; SO_2: sulphur dioxide; SO_4^{2-}: sulfate ion.

Table S4. Relative risks (95%CI) for single-day lag and cumulative-day lag for IS, DM and COPD mortality (lag 4)

lag	SO_4^{2-}	NH_4^+	NO_3^-	OM	BC
IS					
lag 0	1.046 (0.998, 1.097)	1.032 (0.983, 1.085)	1.009 (0.976, 1.044)	1.004 (0.970, 1.039)	1.023 (0.880, 1.191)
lag 1	0.996 (0.946, 1.048)	1.003 (0.956, 1.053)	1.007 (0.976, 1.039)	1.013 (0.977, 1.050)	1.061 (0.901, 1.250)
lag 2	1.017 (0.968, 1.069)	1.015 (0.969, 1.064)	1.010 (0.980, 1.040)	1.004 (0.972, 1.037)	1.028 (0.883, 1.197)
lag 3	0.978 (0.933, 1.026)	0.985 (0.942, 1.031)	0.993 (0.964, 1.022)	0.992 (0.961, 1.023)	0.963 (0.831, 1.116)
lag 4	1.032 (0.992, 1.073)	1.028 (0.990, 1.068)	1.016 (0.991, 1.042)	1.016 (0.990, 1.044)	1.042 (0.918, 1.183)
lag 0-1	1.042 (0.988, 1.099)	1.036 (0.980, 1.096)	1.016 (0.978, 1.056)	1.017 (0.976, 1.060)	1.086 (0.908, 1.300)
lag 0-2	1.060 (1.004, 1.119)	1.052 (0.993, 1.114)	1.026 (0.987, 1.067)	1.021 (0.981, 1.063)	1.117 (0.933, 1.337)
lag 0-3	1.037 (0.980, 1.097)	1.037 (0.976, 1.100)	1.019 (0.978, 1.061)	1.013 (0.971, 1.056)	1.076 (0.890, 1.301)
lag 0-4	1.070 (1.009, 1.135)	1.066 (1.000, 1.135)	1.035 (0.992, 1.081)	1.029 (0.986, 1.075)	1.121 (0.922, 1.363)
COPD					
lag 0	1.066 (1.004, 1.133)	1.060 (0.996, 1.129)	1.027 (0.985, 1.071)	1.031 (0.988, 1.075)	1.224 (1.014, 1.479)
lag 1	0.973 (0.913, 1.038)	0.975 (0.918, 1.036)	0.989 (0.951, 1.028)	1.009 (0.966, 1.054)	0.970 (0.792, 1.187)
lag 2	1.018 (0.956, 1.083)	1.016 (0.959, 1.077)	1.011 (0.974, 1.049)	1.000 (0.961, 1.040)	1.011 (0.838, 1.220)
lag 3	0.973 (0.917, 1.033)	0.966 (0.913, 1.022)	0.977 (0.942, 1.012)	0.990 (0.954, 1.029)	0.964 (0.804, 1.155)
lag 4	1.038 (0.988, 1.090)	1.038 (0.990, 1.089)	1.022 (0.991, 1.053)	1.014 (0.981, 1.047)	1.064 (0.911, 1.244)
lag 0-1	1.038 (0.970, 1.110)	1.034 (0.963, 1.110)	1.016 (0.968, 1.066)	1.039 (0.987, 1.094)	1.187 (0.948, 1.486)
lag 0-2	1.056 (0.986, 1.132)	1.051 (0.978, 1.130)	1.026 (0.977, 1.078)	1.039 (0.988, 1.092)	1.200 (0.959, 1.502)
lag 0-3	1.028 (0.957, 1.105)	1.015 (0.941, 1.094)	1.002 (0.952, 1.055)	1.029 (0.976, 1.085)	1.157 (0.913, 1.466)
lag 0-4	1.067 (0.990, 1.150)	1.054 (0.973, 1.141)	1.024 (0.970, 1.081)	1.043 (0.989, 1.101)	1.231 (0.964, 1.573)
DM					
lag 0	1.031 (0.955, 1.114)	1.015 (0.936, 1.099)	1.006 (0.953, 1.062)	1.034 (0.979, 1.093)	1.136 (0.890, 1.450)
lag 1	0.999 (0.921, 1.085)	1.020 (0.944, 1.102)	1.013 (0.963, 1.065)	1.026 (0.969, 1.086)	1.130 (0.869, 1.469)
lag 2	1.018 (0.940, 1.103)	1.015 (0.941, 1.095)	1.020 (0.972, 1.071)	1.018 (0.966, 1.073)	1.098 (0.857, 1.406)
lag 3	1.047 (0.969, 1.131)	1.046 (0.972, 1.125)	1.022 (0.975, 1.071)	1.030 (0.979, 1.084)	1.085 (0.852, 1.382)
lag 4	0.937 (0.878, 0.999)	0.933 (0.877, 0.994)	0.959 (0.920, 0.998)	0.965 (0.924, 1.009)	0.860 (0.697, 1.060)
lag 0-1	1.031 (0.946, 1.123)	1.035 (0.945, 1.132)	1.019 (0.958, 1.084)	1.061 (0.993, 1.134)	1.284 (0.963, 1.712)
lag 0-2	1.050 (0.961, 1.146)	1.050 (0.958, 1.152)	1.039 (0.976, 1.107)	1.080 (1.012, 1.153)	1.409 (1.055, 1.881)
lag 0-3	1.099 (1.003, 1.204)	1.098 (0.998, 1.209)	1.062 (0.995, 1.135)	1.113 (1.039, 1.191)	1.529 (1.127, 2.074)
lag 0-4	1.029 (0.935, 1.133)	1.025 (0.926, 1.136)	1.018 (0.950, 1.091)	1.074 (1.002, 1.152)	1.314 (0.958, 1.802)

Abbreviations: BC: black carbon; COPD: chronic obstructive pulmonary disease; CI: confidence interval; DM: diabetes mellitus; NH_4^+: ammonium ion; NO_3^-: nitrate ion; OM: organic matter; IS: Ischemic stroke; SD: standard deviation; SO_2: sulphur dioxide; SO_4^{2-}: sulfate ion.

Table S5. Relative risks (95%CI) for single-day lag and cumulative-day lag for IS, DM and COPD mortality (lag 14)

lag	SO_4^{2-}	NH_4^+	NO_3^-	OM	BC
IS					
lag 0	1.045 (1.000, 1.092)	1.032 (0.985, 1.082)	1.010 (0.978, 1.043)	1.007 (0.974, 1.041)	1.038 (0.898, 1.199)
lag 1	1.006 (0.982, 1.031)	1.011 (0.986, 1.036)	1.009 (0.993, 1.027)	1.006 (0.987, 1.025)	1.038 (0.951, 1.133)
lag 2	1.002 (0.984, 1.019)	1.006 (0.989, 1.024)	1.007 (0.995, 1.019)	1.005 (0.992, 1.019)	1.025 (0.965, 1.089)
lag 3	1.006 (0.994, 1.019)	1.008 (0.995, 1.021)	1.005 (0.996, 1.014)	1.005 (0.995, 1.015)	1.014 (0.969, 1.061)
lag 4	1.009 (0.995, 1.023)	1.009 (0.995, 1.024)	1.005 (0.996, 1.015)	1.005 (0.994, 1.015)	1.011 (0.962, 1.061)
lag 5	1.010 (0.998, 1.021)	1.010 (0.999, 1.022)	1.006 (0.998, 1.014)	1.004 (0.996, 1.013)	1.011 (0.973, 1.052)
lag 6	1.010 (1.000, 1.019)	1.011 (1.001, 1.021)	1.007 (1.000, 1.013)	1.004 (0.997, 1.011)	1.013 (0.982, 1.046)
lag 7	1.009 (1.000, 1.019)	1.011 (1.001, 1.021)	1.007 (1.001, 1.014)	1.004 (0.997, 1.010)	1.014 (0.982, 1.048)
lag 8	1.008 (0.999, 1.018)	1.010 (1.000, 1.021)	1.007 (1.000, 1.014)	1.003 (0.996, 1.010)	1.014 (0.981, 1.049)
lag 9	1.007 (0.998, 1.017)	1.009 (0.999, 1.019)	1.007 (1.000, 1.013)	1.003 (0.996, 1.010)	1.013 (0.981, 1.047)
lag 10	1.006 (0.997, 1.015)	1.008 (0.999, 1.017)	1.006 (1.000, 1.012)	1.003 (0.996, 1.009)	1.012 (0.982, 1.043)
lag 11	1.004 (0.995, 1.012)	1.006 (0.998, 1.015)	1.005 (0.999, 1.011)	1.003 (0.997, 1.009)	1.009 (0.981, 1.039)
lag 12	1.002 (0.993, 1.011)	1.005 (0.995, 1.014)	1.004 (0.997, 1.010)	1.002 (0.996, 1.009)	1.007 (0.975, 1.039)
lag 13	1.000 (0.988, 1.012)	1.003 (0.990, 1.015)	1.002 (0.994, 1.011)	1.002 (0.994, 1.011)	1.004 (0.964, 1.045)
lag 14	0.998 (0.983, 1.013)	1.001 (0.985, 1.017)	1.001 (0.990, 1.012)	1.002 (0.991, 1.013)	1.001 (0.948, 1.056)
lag 0-1	1.052 (1.005, 1.101)	1.044 (0.993, 1.097)	1.020 (0.985, 1.055)	1.013 (0.978, 1.049)	1.077 (0.924, 1.256)
lag 0-2	1.053 (1.003, 1.106)	1.050 (0.996, 1.107)	1.027 (0.990, 1.065)	1.018 (0.980, 1.058)	1.104 (0.934, 1.305)
lag 0-3	1.060 (1.006, 1.116)	1.059 (1.001, 1.120)	1.032 (0.993, 1.072)	1.023 (0.984, 1.064)	1.119 (0.940, 1.332)
lag 0-4	1.069 (1.010, 1.131)	1.069 (1.006, 1.135)	1.038 (0.996, 1.081)	1.028 (0.986, 1.071)	1.131 (0.939, 1.363)
lag 0-5	1.079 (1.015, 1.148)	1.080 (1.011, 1.153)	1.044 (0.998, 1.092)	1.032 (0.987, 1.079)	1.144 (0.935, 1.400)
lag 0-6	1.090 (1.020, 1.165)	1.092 (1.017, 1.172)	1.051 (1.002, 1.103)	1.036 (0.988, 1.086)	1.159 (0.934, 1.440)
lag 0-7	1.100 (1.025, 1.181)	1.103 (1.023, 1.190)	1.059 (1.007, 1.114)	1.040 (0.989, 1.093)	1.176 (0.935, 1.480)
lag 0-8	1.109 (1.029, 1.196)	1.115 (1.029, 1.208)	1.067 (1.011, 1.125)	1.043 (0.990, 1.100)	1.193 (0.935, 1.522)
lag 0-9	1.117 (1.031, 1.210)	1.125 (1.034, 1.225)	1.074 (1.015, 1.136)	1.046 (0.990, 1.107)	1.208 (0.933, 1.565)
lag 0-10	1.123 (1.032, 1.222)	1.134 (1.037, 1.241)	1.080 (1.018, 1.147)	1.049 (0.989, 1.113)	1.222 (0.929, 1.608)
lag 0-11	1.128 (1.032, 1.233)	1.142 (1.039, 1.254)	1.086 (1.020, 1.156)	1.052 (0.989, 1.119)	1.234 (0.925, 1.647)
lag 0-12	1.130 (1.029, 1.240)	1.147 (1.040, 1.265)	1.089 (1.021, 1.163)	1.055 (0.989, 1.125)	1.242 (0.918, 1.680)
lag 0-13	1.130 (1.025, 1.246)	1.150 (1.038, 1.274)	1.092 (1.020, 1.169)	1.057 (0.988, 1.131)	1.247 (0.908, 1.712)
lag 0-14	1.128 (1.017, 1.250)	1.151 (1.033, 1.282)	1.093 (1.017, 1.175)	1.059 (0.986, 1.137)	1.247 (0.891, 1.747)
COPD					
lag 0	1.067 (1.009, 1.128)	1.062 (1.001, 1.127)	1.031 (0.990, 1.073)	1.037 (0.996, 1.080)	1.243 (1.038, 1.489)
lag 1	0.980 (0.950, 1.011)	0.979 (0.948, 1.010)	0.987 (0.967, 1.008)	0.994 (0.971, 1.018)	0.950 (0.852, 1.059)

lag	SO_4^{2-}	NH_4^+	NO_3^-	OM	BC
lag 2	0.988 (0.966, 1.009)	0.986 (0.965, 1.008)	0.993 (0.978, 1.007)	0.998 (0.982, 1.014)	0.968 (0.898, 1.044)
lag 3	1.011 (0.995, 1.027)	1.009 (0.992, 1.026)	1.006 (0.995, 1.017)	1.009 (0.997, 1.021)	1.039 (0.982, 1.099)
lag 4	1.016 (0.998, 1.034)	1.014 (0.996, 1.032)	1.009 (0.997, 1.021)	1.011 (0.998, 1.024)	1.056 (0.994, 1.122)
lag 5	1.010 (0.996, 1.025)	1.009 (0.994, 1.024)	1.005 (0.995, 1.015)	1.008 (0.997, 1.018)	1.041 (0.991, 1.093)
lag 6	1.002 (0.990, 1.014)	1.001 (0.989, 1.013)	1.000 (0.992, 1.008)	1.003 (0.995, 1.012)	1.017 (0.978, 1.059)
lag 7	0.996 (0.984, 1.008)	0.996 (0.983, 1.008)	0.997 (0.988, 1.005)	1.000 (0.992, 1.009)	1.000 (0.960, 1.042)
lag 8	0.993 (0.980, 1.005)	0.992 (0.979, 1.005)	0.995 (0.986, 1.003)	0.998 (0.990, 1.008)	0.990 (0.949, 1.033)
lag 9	0.991 (0.979, 1.003)	0.991 (0.978, 1.003)	0.994 (0.986, 1.002)	0.998 (0.989, 1.007)	0.984 (0.944, 1.026)
lag 10	0.991 (0.980, 1.003)	0.991 (0.979, 1.003)	0.994 (0.987, 1.002)	0.998 (0.990, 1.006)	0.983 (0.946, 1.021)
lag 11	0.993 (0.982, 1.003)	0.992 (0.981, 1.003)	0.995 (0.988, 1.003)	0.999 (0.992, 1.007)	0.985 (0.950, 1.022)
lag 12	0.995 (0.983, 1.007)	0.995 (0.982, 1.007)	0.997 (0.989, 1.005)	1.001 (0.992, 1.009)	0.990 (0.951, 1.031)
lag 13	0.998 (0.983, 1.013)	0.997 (0.982, 1.013)	0.999 (0.989, 1.010)	1.003 (0.992, 1.014)	0.997 (0.947, 1.049)
lag 14	1.002 (0.982, 1.021)	1.001 (0.981, 1.021)	1.002 (0.988, 1.015)	1.005 (0.991, 1.019)	1.004 (0.939, 1.074)
lag 0-1	1.045 (0.986, 1.108)	1.040 (0.976, 1.107)	1.017 (0.974, 1.062)	1.031 (0.987, 1.078)	1.181 (0.975, 1.432)
lag 0-2	1.032 (0.970, 1.098)	1.025 (0.959, 1.096)	1.010 (0.965, 1.057)	1.029 (0.982, 1.079)	1.144 (0.928, 1.409)
lag 0-3	1.044 (0.977, 1.114)	1.034 (0.964, 1.110)	1.016 (0.968, 1.066)	1.038 (0.989, 1.090)	1.188 (0.955, 1.478)
lag 0-4	1.060 (0.987, 1.138)	1.049 (0.972, 1.132)	1.025 (0.973, 1.079)	1.050 (0.997, 1.105)	1.255 (0.993, 1.584)
lag 0-5	1.071 (0.990, 1.158)	1.058 (0.974, 1.150)	1.030 (0.974, 1.089)	1.058 (1.001, 1.118)	1.306 (1.014, 1.682)
lag 0-6	1.073 (0.986, 1.167)	1.059 (0.969, 1.158)	1.030 (0.970, 1.094)	1.062 (1.001, 1.126)	1.328 (1.013, 1.742)
lag 0-7	1.069 (0.977, 1.169)	1.055 (0.959, 1.160)	1.027 (0.963, 1.094)	1.062 (0.998, 1.130)	1.329 (0.996, 1.773)
lag 0-8	1.061 (0.964, 1.167)	1.046 (0.946, 1.157)	1.021 (0.955, 1.092)	1.060 (0.993, 1.132)	1.316 (0.969, 1.786)
lag 0-9	1.051 (0.950, 1.163)	1.037 (0.932, 1.154)	1.015 (0.945, 1.090)	1.058 (0.987, 1.134)	1.295 (0.935, 1.792)
lag 0-10	1.042 (0.936, 1.160)	1.027 (0.918, 1.150)	1.009 (0.936, 1.088)	1.056 (0.981, 1.137)	1.273 (0.902, 1.797)
lag 0-11	1.034 (0.924, 1.158)	1.019 (0.906, 1.147)	1.005 (0.929, 1.087)	1.055 (0.977, 1.140)	1.254 (0.872, 1.804)
lag 0-12	1.029 (0.915, 1.158)	1.014 (0.896, 1.147)	1.002 (0.923, 1.087)	1.056 (0.974, 1.145)	1.242 (0.848, 1.818)
lag 0-13	1.027 (0.908, 1.163)	1.011 (0.889, 1.151)	1.001 (0.919, 1.091)	1.059 (0.973, 1.152)	1.238 (0.830, 1.846)
lag 0-14	1.029 (0.903, 1.173)	1.012 (0.883, 1.160)	1.003 (0.916, 1.098)	1.064 (0.973, 1.163)	1.243 (0.813, 1.900)
DM					
lag 0	1.015 (0.945, 1.090)	1.001 (0.928, 1.080)	0.995 (0.945, 1.049)	1.030 (0.976, 1.086)	1.119 (0.886, 1.414)
lag 1	1.041 (1.001, 1.083)	1.051 (1.010, 1.093)	1.037 (1.010, 1.066)	1.043 (1.012, 1.076)	1.215 (1.055, 1.398)
lag 2	1.017 (0.989, 1.046)	1.022 (0.994, 1.051)	1.017 (0.998, 1.036)	1.021 (0.999, 1.043)	1.096 (0.994, 1.208)
lag 3	0.993 (0.973, 1.014)	0.990 (0.969, 1.011)	0.992 (0.978, 1.007)	0.999 (0.984, 1.015)	0.988 (0.918, 1.064)
lag 4	0.988 (0.966, 1.010)	0.981 (0.959, 1.004)	0.986 (0.971, 1.001)	0.992 (0.976, 1.010)	0.958 (0.885, 1.038)
lag 5	0.993 (0.975, 1.011)	0.987 (0.969, 1.006)	0.990 (0.978, 1.003)	0.994 (0.981, 1.008)	0.970 (0.910, 1.034)
lag 6	1.000 (0.985, 1.015)	0.996 (0.981, 1.012)	0.997 (0.987, 1.008)	0.999 (0.988, 1.010)	0.994 (0.944, 1.047)

lag	SO_4^{2-}	NH_4^+	NO_3^-	OM	BC
lag 7	1.006 (0.991, 1.021)	1.004 (0.988, 1.020)	1.002 (0.992, 1.013)	1.003 (0.991, 1.014)	1.014 (0.962, 1.068)
lag 8	1.010 (0.994, 1.026)	1.008 (0.992, 1.025)	1.006 (0.995, 1.017)	1.005 (0.993, 1.017)	1.028 (0.974, 1.085)
lag 9	1.012 (0.996, 1.027)	1.011 (0.995, 1.027)	1.008 (0.997, 1.019)	1.007 (0.995, 1.018)	1.037 (0.984, 1.094)
lag 10	1.012 (0.998, 1.027)	1.012 (0.997, 1.027)	1.008 (0.998, 1.018)	1.008 (0.997, 1.018)	1.043 (0.993, 1.095)
lag 11	1.012 (0.998, 1.026)	1.011 (0.997, 1.025)	1.008 (0.998, 1.017)	1.008 (0.998, 1.018)	1.044 (0.997, 1.094)
lag 12	1.010 (0.995, 1.026)	1.009 (0.994, 1.025)	1.006 (0.996, 1.017)	1.008 (0.997, 1.019)	1.044 (0.992, 1.098)
lag 13	1.008 (0.989, 1.028)	1.007 (0.987, 1.027)	1.004 (0.991, 1.018)	1.007 (0.993, 1.021)	1.041 (0.975, 1.112)
lag 14	1.006 (0.982, 1.031)	1.004 (0.979, 1.030)	1.002 (0.985, 1.020)	1.007 (0.988, 1.025)	1.038 (0.953, 1.131)
lag 0-1	1.056 (0.981, 1.137)	1.052 (0.970, 1.140)	1.033 (0.977, 1.092)	1.074 (1.015, 1.137)	1.360 (1.063, 1.739)
lag 0-2	1.074 (0.993, 1.162)	1.075 (0.987, 1.170)	1.050 (0.991, 1.114)	1.097 (1.032, 1.165)	1.490 (1.141, 1.945)
lag 0-3	1.067 (0.981, 1.160)	1.064 (0.972, 1.164)	1.043 (0.980, 1.109)	1.096 (1.029, 1.167)	1.472 (1.115, 1.945)
lag 0-4	1.054 (0.962, 1.154)	1.044 (0.947, 1.151)	1.028 (0.962, 1.099)	1.088 (1.018, 1.162)	1.411 (1.046, 1.902)
lag 0-5	1.046 (0.946, 1.156)	1.030 (0.926, 1.146)	1.018 (0.947, 1.094)	1.081 (1.007, 1.162)	1.368 (0.988, 1.895)
lag 0-6	1.046 (0.939, 1.165)	1.027 (0.916, 1.152)	1.015 (0.940, 1.097)	1.080 (1.001, 1.166)	1.360 (0.958, 1.930)
lag 0-7	1.052 (0.938, 1.180)	1.031 (0.912, 1.164)	1.017 (0.937, 1.104)	1.083 (0.999, 1.174)	1.379 (0.951, 1.999)
lag 0-8	1.063 (0.940, 1.201)	1.039 (0.913, 1.183)	1.023 (0.938, 1.116)	1.088 (0.999, 1.185)	1.417 (0.956, 2.102)
lag 0-9	1.075 (0.944, 1.224)	1.050 (0.916, 1.205)	1.031 (0.941, 1.130)	1.096 (1.001, 1.199)	1.470 (0.968, 2.234)
lag 0-10	1.088 (0.949, 1.248)	1.062 (0.919, 1.228)	1.040 (0.944, 1.145)	1.104 (1.004, 1.215)	1.533 (0.984, 2.387)
lag 0-11	1.101 (0.953, 1.272)	1.074 (0.923, 1.250)	1.047 (0.946, 1.159)	1.113 (1.007, 1.230)	1.601 (1.004, 2.552)
lag 0-12	1.112 (0.957, 1.294)	1.084 (0.925, 1.271)	1.054 (0.948, 1.172)	1.122 (1.011, 1.245)	1.671 (1.025, 2.722)
lag 0-13	1.122 (0.958, 1.314)	1.091 (0.924, 1.288)	1.058 (0.948, 1.182)	1.130 (1.013, 1.260)	1.740 (1.043, 2.903)
lag 0-14	1.128 (0.955, 1.333)	1.096 (0.919, 1.306)	1.061 (0.944, 1.192)	1.138 (1.014, 1.276)	1.806 (1.049, 3.109)

Abbreviations: BC: black carbon; COPD: chronic obstructive pulmonary disease; CI: confidence interval; DM: diabetes mellitus; NH_4^+: ammonium ion; NO_3^-: nitrate ion; OM: organic matter; IS: Ischemic stroke; SD: standard deviation; SO_2: sulphur dioxide; SO_4^{2-}: sulfate ion.

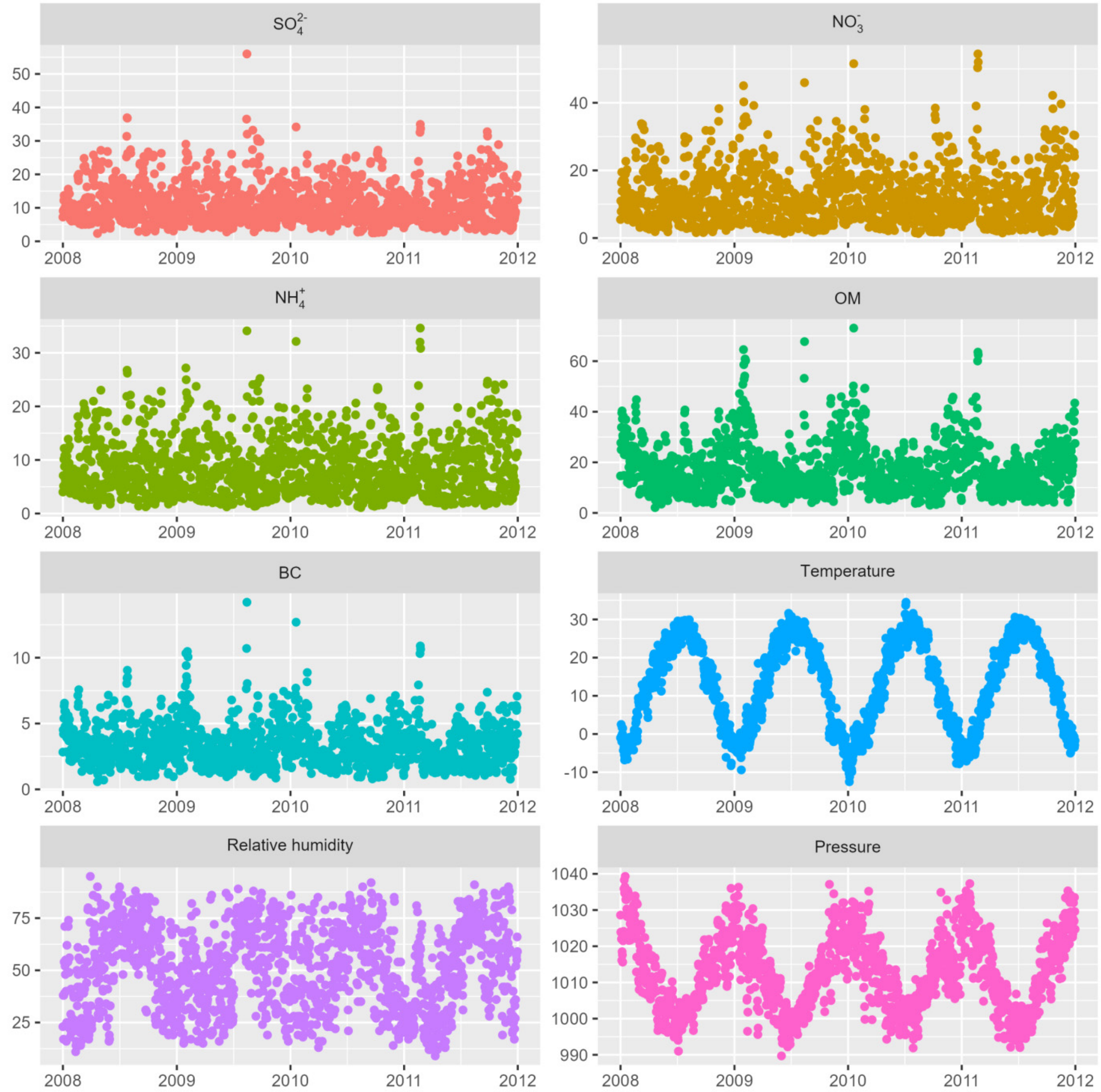

Figure S1. Daily concentrations of air pollutants and meteorological factors between 2008 and 2011

Abbreviations: BC: black carbon; NH_4^+: ammonium ion; NO_3^-: nitrate ion; OM: organic matter; SO_4^{2-}: sulfate ion.

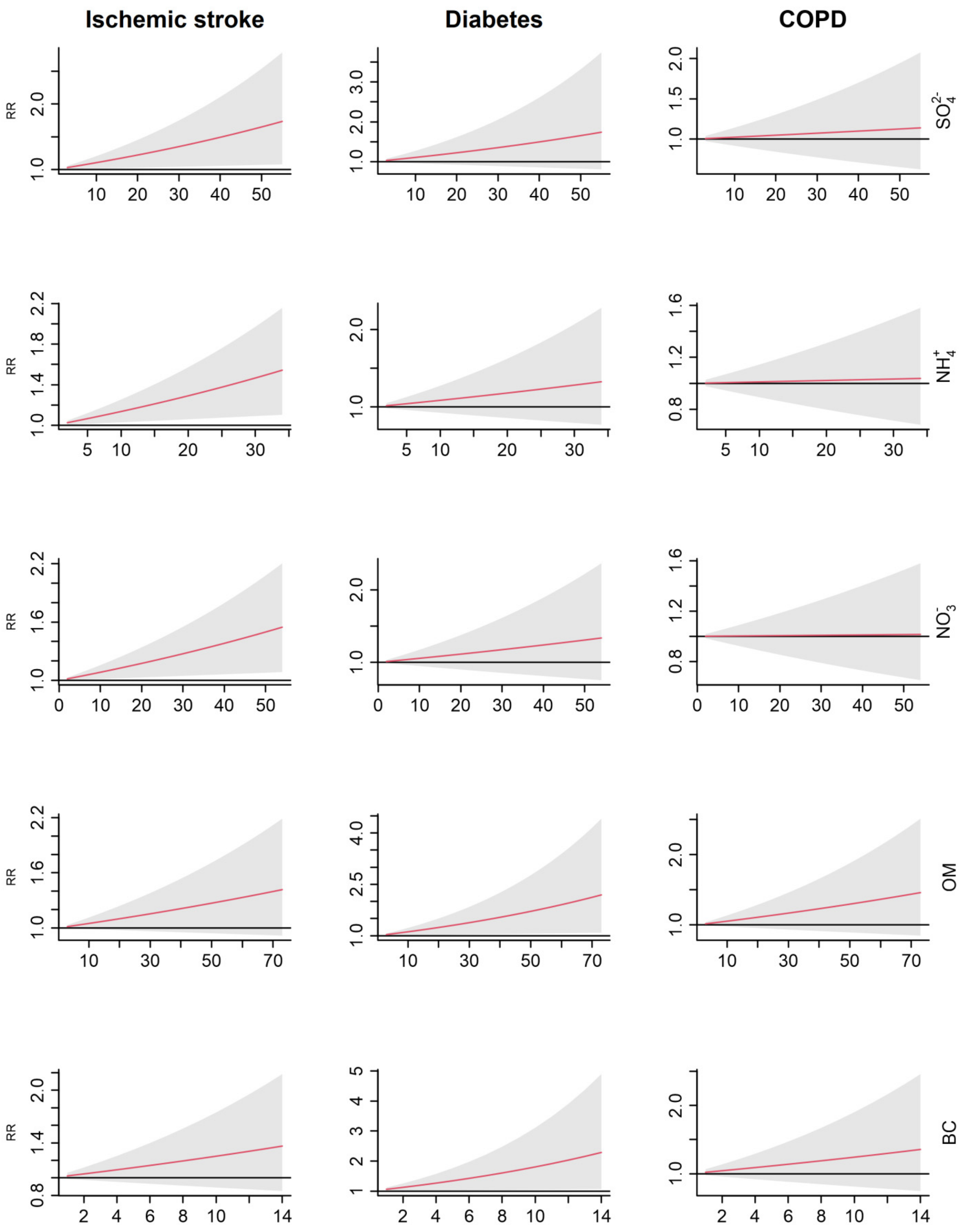

Figure S2. Exposure-response relationships for SO_4^{2-}, NH_4^+, NO_3^-, OM and BC (lag 0-14 days) with Ischemic stroke, Diabetes, and COPD mortality

Abbreviations: BC: black carbon; COPD: chronic obstructive pulmonary disease; NH_4^+: ammonium ion; NO_3^-: nitrate ion; OM: organic matter; RR: Relative risks; SO_4^{2-}: sulfate ion.

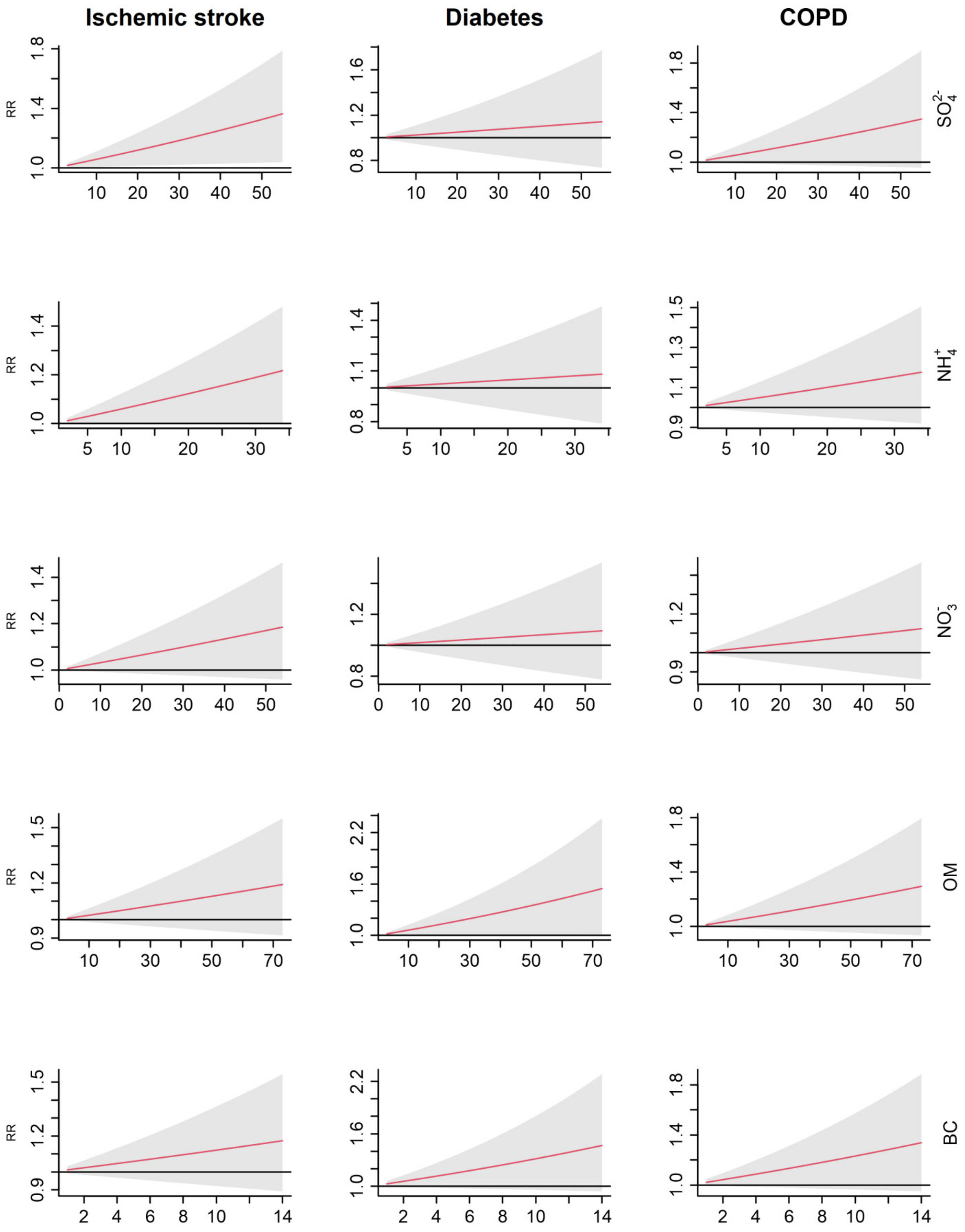

Figure S3. Exposure-response relationships for SO_4^{2-}, NH_4^+, NO_3^-, OM and BC (lag 0-4 days) with Ischemic stroke, Diabetes, and COPD mortality

Abbreviations: BC: black carbon; COPD: chronic obstructive pulmonary disease; NH_4^+: ammonium ion; NO_3^-: nitrate ion; OM: organic matter; RR: Relative risks; SO_4^{2-}: sulfate ion.

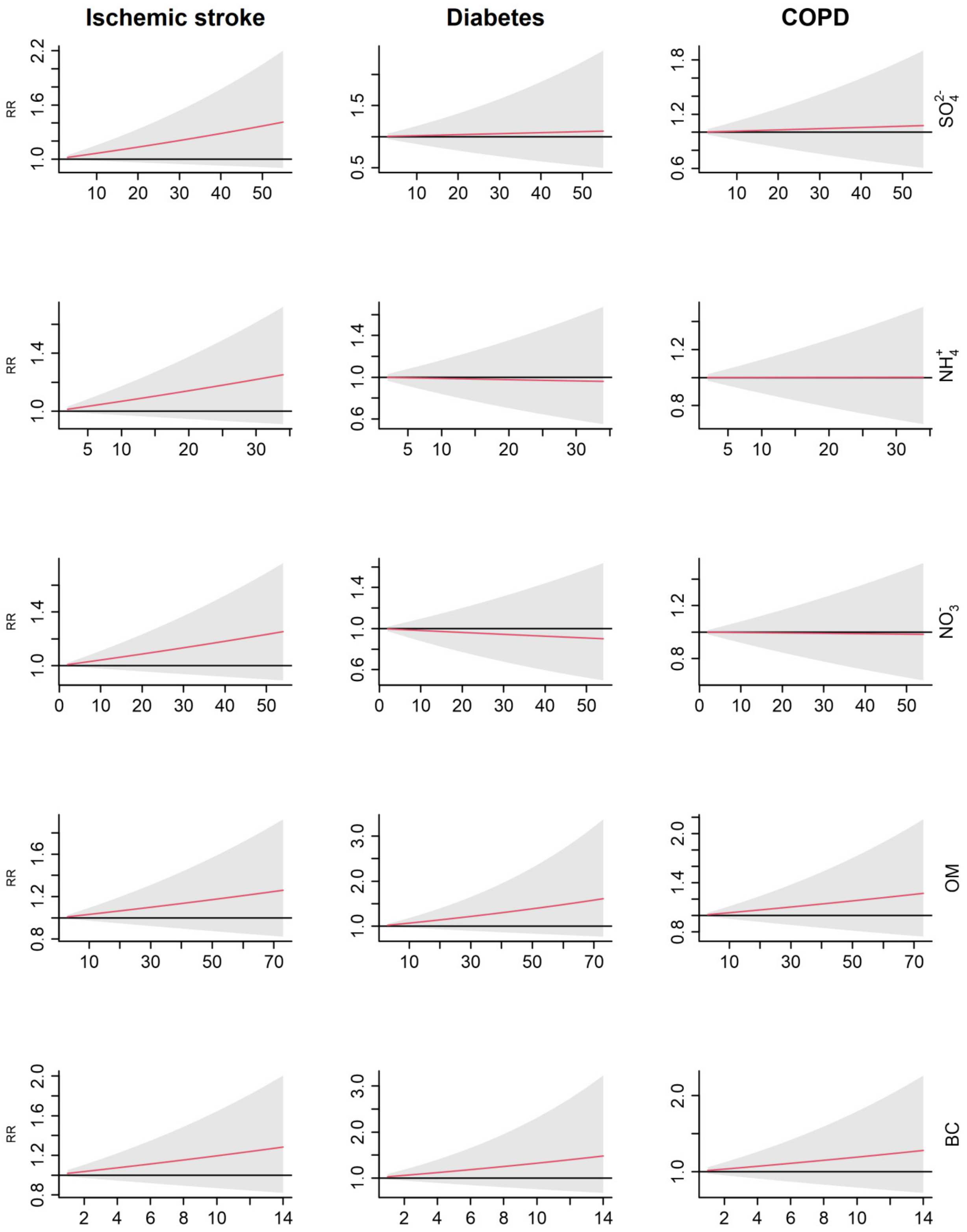

Figure S4. Exposure-response relationships for SO_4^{2-}, NH_4^+, NO_3^-, OM and BC (lag 0-7 days) with Ischemic stroke, Diabetes, and COPD mortality in males

Abbreviations: BC: black carbon; COPD: chronic obstructive pulmonary disease; NH_4^+: ammonium ion; NO_3^-: nitrate ion; OM: organic matter; RR: Relative risks; SO_4^{2-}: sulfate ion.

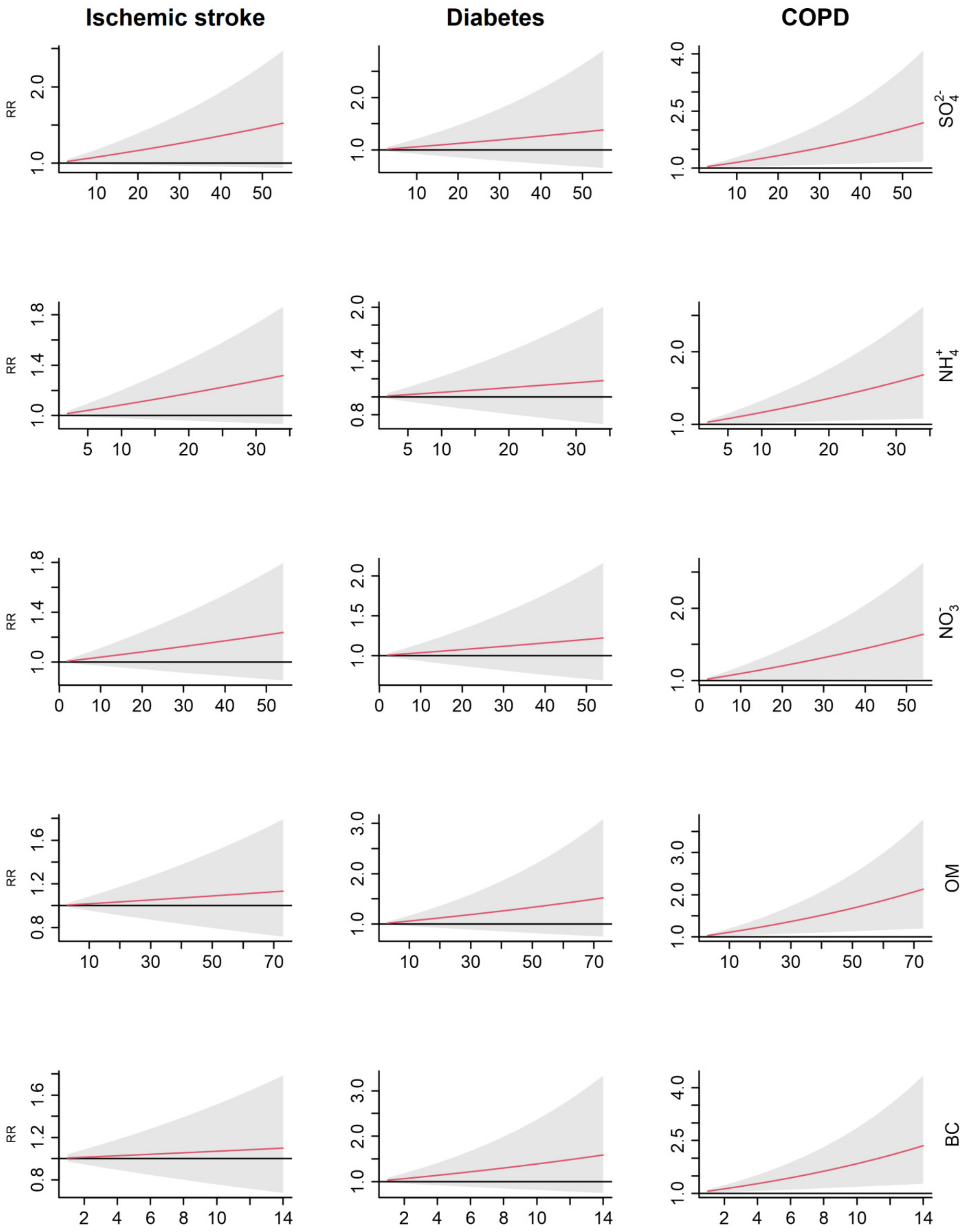

Figure S5. Exposure-response relationships for SO_4^{2-}, NH_4^+, NO_3^-, OM and BC (lag 0-7 days) with Ischemic stroke, Diabetes, and COPD mortality in females

Abbreviations: BC: black carbon; COPD: chronic obstructive pulmonary disease; NH_4^+: ammonium ion; NO_3^-: nitrate ion; OM: organic matter; RR: Relative risks; SO_4^{2-}: sulfate ion.

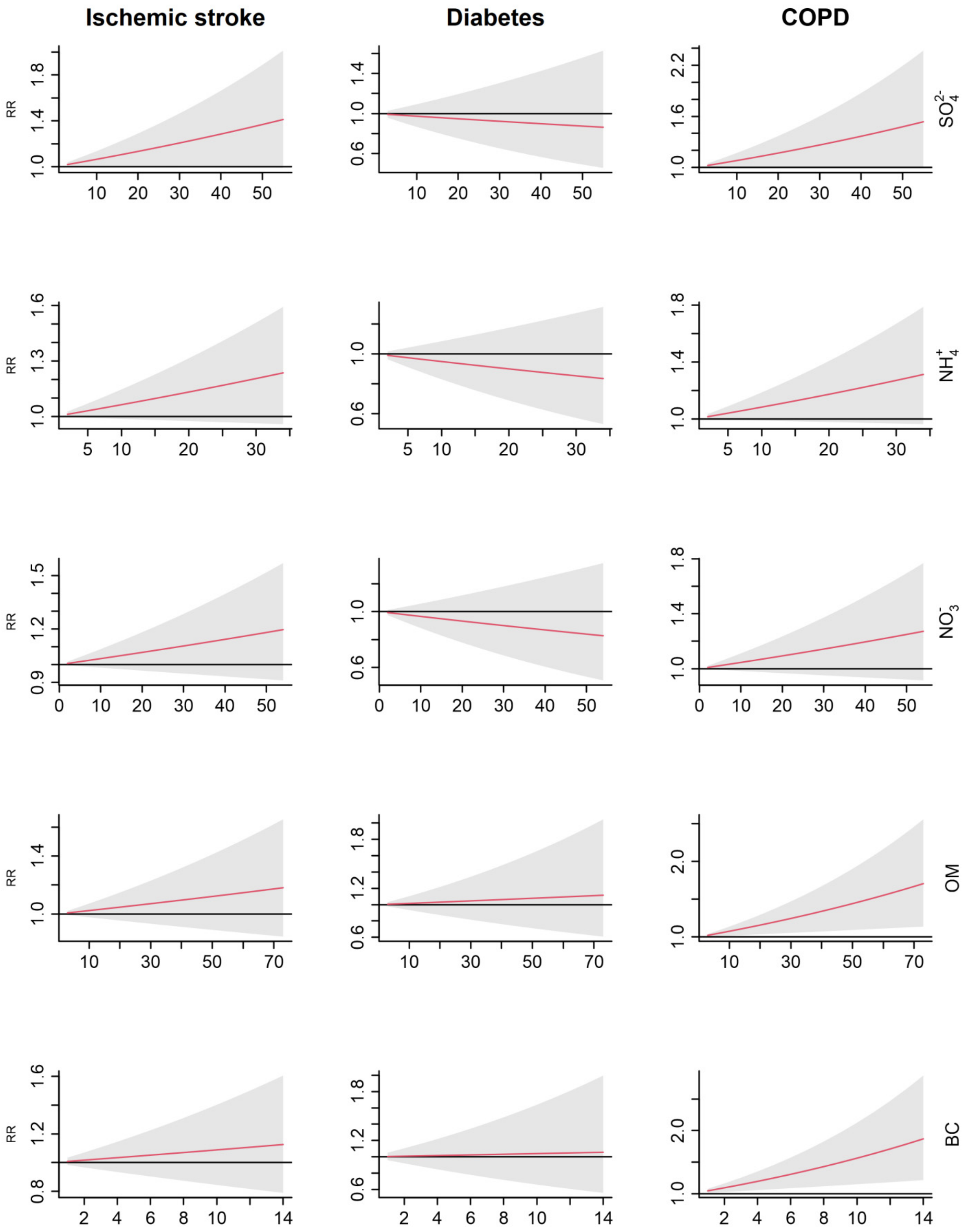

Figure S6. Exposure-response relationships for SO_4^{2-}, NH_4^+, NO_3^-, OM and BC (lag 0-7 days) with Ischemic stroke, Diabetes, and COPD mortality in the older population (age ≥ 65 years old)

Abbreviations: BC: black carbon; COPD: chronic obstructive pulmonary disease; NH_4^+: ammonium ion; NO_3^-: nitrate ion; OM: organic matter; RR: Relative risks; SO_4^{2-}: sulfate ion.

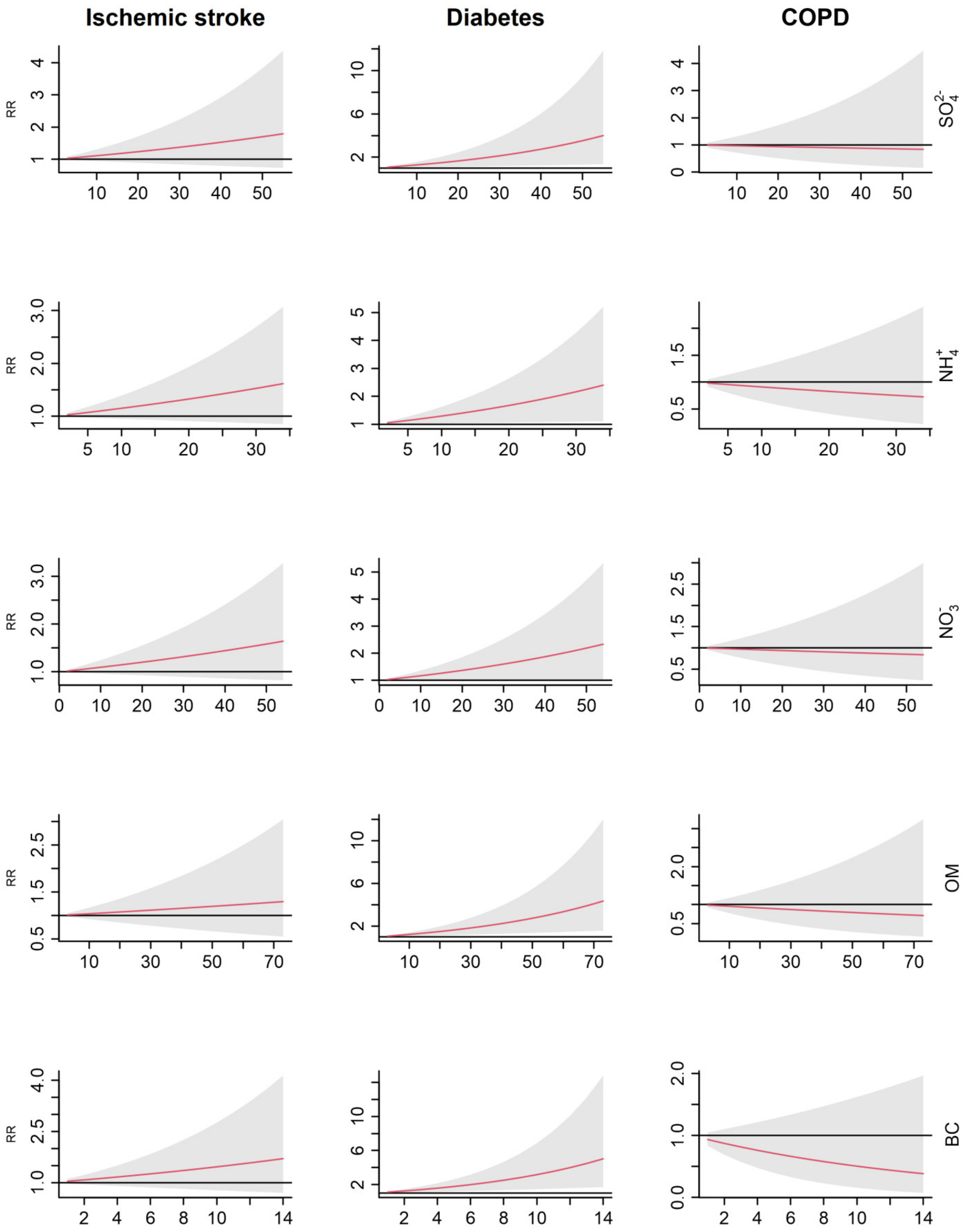

Figure S7. Exposure-response relationships for SO_4^{2-}, NH_4^+, NO_3^-, OM and BC (lag 0-7 days) with Ischemic stroke, Diabetes, and COPD mortality in the younger population (age < 65 years old)

Abbreviations: BC: black carbon; COPD: chronic obstructive pulmonary disease; NH_4^+: ammonium ion; NO_3^-: nitrate ion; OM: organic matter; RR: Relative risks; SO_4^{2-}: sulfate ion.
